# The V-ATPase a2 isoform controls mammary gland development through Notch and TGF-*β* signaling

**DOI:** 10.1038/cddis.2016.347

**Published:** 2016-11-03

**Authors:** Sahithi Pamarthy, Liquin Mao, Gajendra K Katara, Sara Fleetwood, Arpita Kulshreshta, Alice Gilman-Sachs, Kenneth D Beaman

**Affiliations:** 1 Department of Microbiology and Immunology, Rosalind Franklin University of Medicine and Science, North Chicago, IL 60064, USA; 2 Division of Hematology and Oncology, Feinberg School of Medicine, Northwestern University, Chicago, IL 60611, USA

## Abstract

Among all tissues and organs, the mammary gland is unique because most of its development occurs in adulthood. Notch signaling has a major role in mammary gland development and has been implicated in breast cancer. The vacuolar-ATPase (V-ATPase) is a proton pump responsible for the regulation and control of pH in intracellular vesicles and the extracellular milieu. We have previously reported that a2V-ATPase (a2V), an isoform of ‘a' subunit of V-ATPase, regulates processing of Notch receptor and alters Notch signaling in breast cancer. To study the role of a2V in mammary gland development, we generated an a2V-KO model (conditional mammary knockout a2V mouse strain). During normal mammary gland development, the basal level expression of a2V increased from puberty, virginity, and pregnancy through the lactation stage and then decreased during involution. Litters of a2V-KO mice weighed significantly less when compared with litters from wild-type mice and showed reduced expression of the lactation marker *β*-casein. Whole-mount analysis of mammary glands demonstrated impaired ductal elongation and bifurcation in a2V-KO mice. Consequently, we found disintegrated mammary epithelium as seen by basal and luminal epithelial staining, although the rate of proliferation remained unchanged. Delayed mammary morphogenesis in a2V-KO mice was associated with aberrant activation of Notch and TGF-*β* (transforming growth factor-*β*) pathways. Notably, Hey1 (hairy/enhancer-of-split related with YRPW motif) and Smad2, the key downstream mediators of Notch and TGF-*β* pathways, respectively, were upregulated in a2V-KO mice and also in human mammary epithelial cells treated with a2V siRNA. Taken together, our results show that a2V deficiency disrupts the endolysosomal route in Notch and TGF signaling, thereby impairing mammary gland development. Our findings have broader implications in developmental and oncogenic cellular environments where V-ATPase, Notch and TGF-*β* are crucial for cell survival.

The mammary gland is one of the few organs whose development occurs postnatally. The gland is transformed from a nonfunctional organ into a mature, milk-producing gland evolved to nurture the newborn.^1^ The adult mammary gland mainly consists of epithelial cells surrounded by adipocytes, vascular endothelial cells, stromal cells and immune cells. The mammary epithelium forms the main ductal network of the bilayered gland that consists of an outer layer of myoepithelial cells and inner layer of luminal epithelial cells. Although it is the mammary epithelium that is the most proliferative as it invades the fat pad, the surrounding mammary stroma provides important signals to influence epithelial cell fate. Taken together, alterations in breast biology during reproductive life influence breast cancer risk.^2^ Mammary gland development is initiated at the embryonic stage, but expansion only begins at puberty. After the initial development during puberty, the mammary gland is extensively remodeled during pregnancy. Finally, there is a postlactational regression or involution of the gland and after pregnancy it returns to a structural state similar to the virgin gland. Signaling pathways that initiate and regulate lineage commitment tightly coordinate the series of growth and involution stages.^3, 4^

Notch signaling has a major role in development and has been implicated in most cancers.^5^ In mammals there are four Notch receptors (Notch 1–4) and five ligands (Jagged1, 2; Delta-like 1, 3 and 4). Upon ligand-receptor binding, the Notch receptor's intracellular domain (NICD) is cleaved and translocated to the nucleus, resulting in the activation of target genes that are involved in cell fate determination and proliferation.^6^ In the mammary gland, Notch promotes mammary epithelial stem cell activity and their commitment towards luminal lineage, and the aberrant activation of the Notch pathway results in an altered lineage.^7, 8, 9^ Furthermore, the heterogeneous phenotypes of Notch signaling in mammary development and tumorigenesis are attributed to dose-dependent Notch activation.^10^

Transforming growth factor-*β* (TGF-*β*) is a pluripotent cytokine with crucial roles in embryonic development and cell fate determination. TGF-*β* signaling has been implicated in mammary gland morphogenesis and breast cancer.^11, 12, 13^ Notch and TGF pathways have similar patterns of expression and spatiotemporal regulation during development and recent reports suggest a direct link between them.^14, 15^ Notably, the endolysosomal pathway is crucial for the activation and degradation of Notch and TGF pathway mediators.^16, 17^

The vacuolar-ATPase (V-ATPase) is responsible for acidification of endosomes and lysosomes in normal cells and is translocated to the plasma membrane of specialized cells such as renal intercalated cells, osteoclasts and most cancer cells, where they acidify the extracellular milieu.^18^ Although reports suggest the involvement of V-ATPase in breast cancer,^19, 20^ its specific role in mammary gland development has not been studied. Structurally, V-ATPase is an ATP-driven multisubunit proton pump consisting of a cytoplasmic V1 domain for ATP hydrolysis and a membrane intrinsic V0 domain composed of a proton-translocating channel with six subunits (a, c, c′, c″, d and e). The ‘a' subunit is of critical importance to the assembly and functioning of V-ATPase.^21^ In mammals, there are four defined isoforms of the ‘a' subunit – a1, a2, a3 and a4, which are responsible for assembly and targeting of V-ATPase to cell surface and to specific organelles within the cell. a2V-ATPase is an ‘a2' subunit containing isoform of V-ATPase (a2V). a2V is a key factor in pregnancy and cancer through its role in autophagy and immunomodulation.^19, 22^ Notably, genetic mutations in human a2V gene result in Cutis Laxa syndrome where patients present with wrinkly skin because of impaired protein glycosylation and elevated TGF-*β* signaling.^23^ Recent reports suggest the involvement of V-ATPase in Notch pathway regulation in *Drosophila* as well as mammalian cells.^24, 25^ We have previously reported that a2V regulates lysosomal degradation of Notch receptor Notch 1 and alters Notch signaling in breast cancer.^26^ Similarly, a2V regulates Notch signaling in inflammation-induced preterm labor in mice.^27^ However, the involvement of a2V in context of Notch and TGF-*β* pathways in mammary gland development has not been explored.

We hypothesized that a2V regulates Notch signaling in addition to its direct involvement in mammary gland development. To investigate our hypothesis, we used an a2V mammary gland knockout (KO) mouse model and show that a2V deficiency results in mammary gland anomalies with disintegrated epithelia, impaired ductal branching and finally lactation defects. Delayed ductal morphogenesis in a2V-KO mice was mediated by aberrant activation of Notch and of TGF-*β* signaling. Similarly, *in vitro* knockdown (KD) of a2V in human mammary epithelial cells (HMEpCs) also resulted in alterations in Notch and TGF-*β* activity. Taken together, these results suggest that a2V has a key role in the mammary gland and identify a novel role for a2V in regulating Notch signaling during development.

## Results

### Expression of a2V during mammary gland development

**Figure 1:**
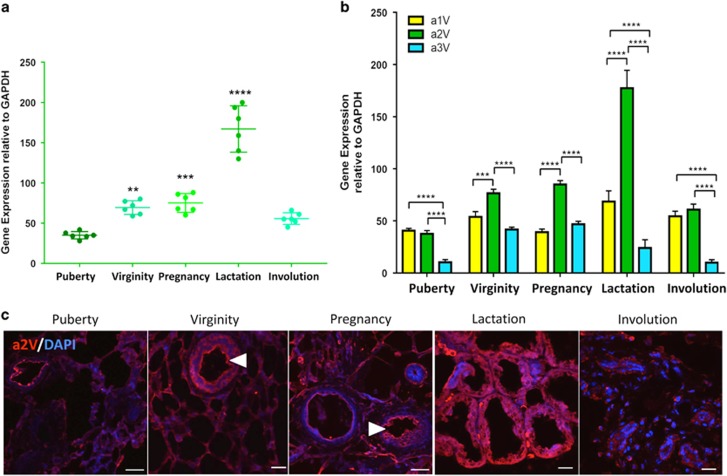
a2V expression during normal mammary development. Inguinal mammary glands from puberty, virginity, pregnancy, lactation and involution stages were harvested and analyzed for gene expression and cellular distribution of a2V. Total RNA extracted from mammary glands was subjected to quantitative real-time-PCR (qRT-PCR) analysis. (**a**) Gene expression of V-ATPase ‘a2' subunit isoform during various stages of gland development compared with puberty. (**b**) Relative gene expression of V-ATPase a1, a2 and a3 subunit isoforms compared with each other at every stage. Glyceraldehyde 3-phosphate dehydrogenase (GAPDH) served as endogenous housekeeping control. Data represent mean±S.E., *n*=6. *P≤0.05, **P≤0.01, ***P≤0.001 and ****P≤0.0001. (**c**) Representative z-stack images from a confocal laser-scanning microscope show expression pattern of V-ATPase ‘a2' subunit isoform (a2V in red). Nucleus was stained with DAPI (diamidino-2-phenylindole; blue). Scale bars: 20 *μ*m

To investigate the role of V-ATPase in mammary gland development, we first evaluated steady-state expression of a2V. We collected mammary glands at five different stages of gland development (puberty, virginity, pregnancy, lactation and involution) and performed real-time PCR of a2V. The relative levels of a2V expression increased through puberty, virginity, pregnancy and lactation and then decreased during involution. During lactation, a2V expression was at significantly higher levels than at other times tested (Figure 1a). Fold increase of gene expressions of epithelial markers keratin 5, keratin 8 and a2V show a robust increase of a2V from pubertal to lactation stage (Supplementary Figure S1b). The tissue-specific expression and cell-type-specific functions of V-ATPase are regulated by the ‘a' subunit isoforms.^28^ In the mammary gland, a2V is most abundantly expressed at all stages followed by the a1 isoform. The a3 isoform was lowest, whereas a4 remained undetected with PCR (Figure 1b). In line with PCR data, confocal microscopy revealed abundant expression of a2V during lactation (Figure 1c). Previous reports have highlighted the role of plasma membrane V-ATPase in acidification of luminal compartment by renal intercalated cells, osteoclasts and epididymal clear cells.^29^ Notably, in the mammary gland, we found plasma membrane expression of a2V in luminal epithelial cells in virgin adult and pregnant mice (Figure 1c, arrowheads). Taken together, these results suggest that a2V is most abundant during lactation.

### Generation of mammary a2V-KO mice

**Figure 2:**
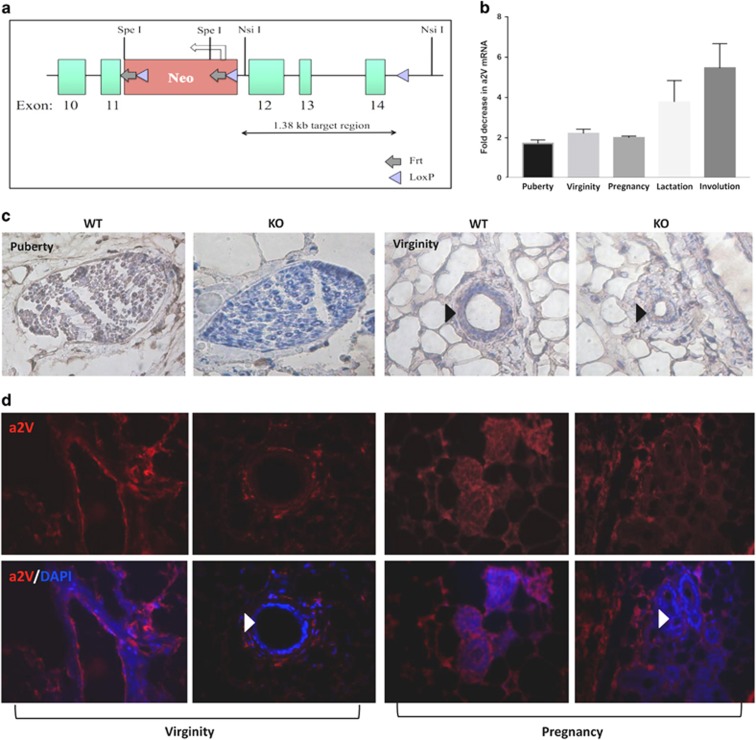
Generation of conditional mammary a2V-KO mice. (**a**) Conditional KO schematic for a2V (gene name ATP6V0a2) is shown. Gene-targeting vector contained exons 10–18 of a2V, of which 12–14 are depicted (green boxes). The LoxP-FRT neo cassette was inserted upstream of exon 12 (red box). A single LoxP site was inserted downstream of exon 14 in the intronic sequence as shown. The schematic also indicates location of key restriction enzyme sites. To assess the KO efficiency, 2nd and 4th mammary glands were dissected from WT and a2V-KO mice. (**b**) Fold decrease in mRNA expression levels of a2V was assessed by real-time-PCR (RT-PCR). before fold-change calculation, the values were normalized to endogenous control glyceraldehyde 3-phosphate dehydrogenase (GAPDH). Data represent mean±S.E.M., *n*=6. Representative images of (**c**) immunohistochemistry of WT and a2V-KO pubertal mammary gland sections and (**d**) immunofluorescence of mammary glands from WT and KO mice from virginity and pregnancy stages stained for a2V protein highlighting efficient KO of a2V in mammary epithelial cells. Brown staining – DAB (3,3' diaminobenzidine); counterstain – hematoxylin. In immunofluorescence, a2V in red and nucleus in blue. Original magnification: × 400

To better understand the physiological functions of a2V in mammary gland development, we generated a mouse model using Cre-LoxP conditional KO strategy. A gene-targeting vector spanning the exons 12–14 of mouse a2V was flanked by LoxP sites (Figure 2a) and used to obtain mice bearing floxed a2V (f) alleles a2V^f/f^, which were bred with MMTV-Cre transgenic mice to obtain a2V conditional mammary KO mice. To detect Cre-mediated a2V deletion, we performed genomic PCR using primers for the LoxP sites, which confirmed genomic deletion of a2V (Supplementary Figure S1b). RT-PCR confirmed efficient KO in mammary gland tissues of a2V-KO mice compared to wild-type (WT) mice (Figure 2b). Compensatory upregulation of ‘a' subunit isoforms in response to depletion of V-ATPase ‘a' subunit has been reported previously.^30^ In a2V-KO mice, we found a slight but not statistically significant upregulation of the a1 isoform at the mRNA level (data not shown). Immunohistochemistry of pubertal and virgin adult mammary glands (Figure 2c) and immunofluorescence of mammary glands from virgin and pregnant mice (Figure 2d, white arrows) revealed a2V protein absence, specifically in the mammary epithelial cells of KO mice (Figure 2c and d). Black arrows in Figure 2c point to the constricted mammary epithelium, which is discussed further in Figures 5 and 6.

### Lactation defects in a2V-KO mice

**Figure 3:**
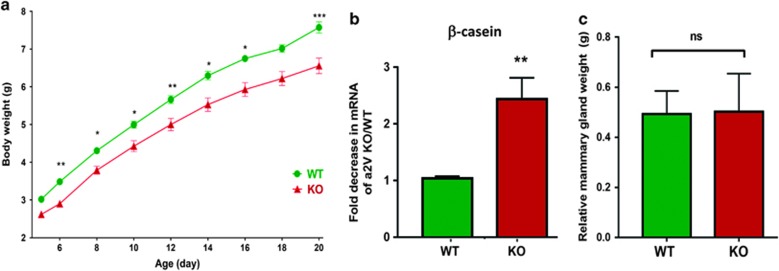
a2V is essential for the lactating mammary gland. (**a**) Growth-curve analysis of litters from WT and KO mice. Mice aged 6–8 weeks were mated and litter sizes ranging from five to eight pups were evaluated. Data from 8-size litter is shown. Pups were weighed during the indicated time points on X axis. Y axis represents body weight in grams. Data represent mean±S.E., *n*=24. (**b**) Real-time-PCR (RT-PCR) showing fold decrease in *β*-casein mRNA from a2V-KO over WT mice. Before fold-change calculation, the values were normalized to endogenous control glyceraldehyde 3-phosphate dehydrogenase (GAPDH). (**c**) Before tissue dissection and harvesting, mammary glands were weighed and normalized to their body weights. Data represent mean±S.E., *n*=6. NS, nonsignificant. *P≤0.05, **P≤0.01 and ***P≤0.001

Abundant expression of a2V during lactation (Figure 1c) led us to further investigate the role of a2V in lactating mammary glands. a2V-KO mice had normal litter sizes and nursing patterns. However, the litters of a2V-KO mice had lower body weight than the litters of their WT counterparts, suggesting a lack of nutrition (Figure 3a). Next, we found that a2V-KO mice displayed a decrease in the expression of *β*-casein, a lactation marker, when compared with WT mice (Figure 3b). We further analyzed and compared body and mammary gland weights of a2V-KO with WT mice. When corrected for body weights, the relative mammary gland weights were similar in a2V-KO and WT in virgin adult mice (Figure 3c) and at all points assessed (data not shown). These findings support the hypothesis that a2V deficiency results in lactation defects and reduced milk protein synthesis.

### Impaired mammary gland morphogenesis in a2V-KO mice

**Figure 4:**
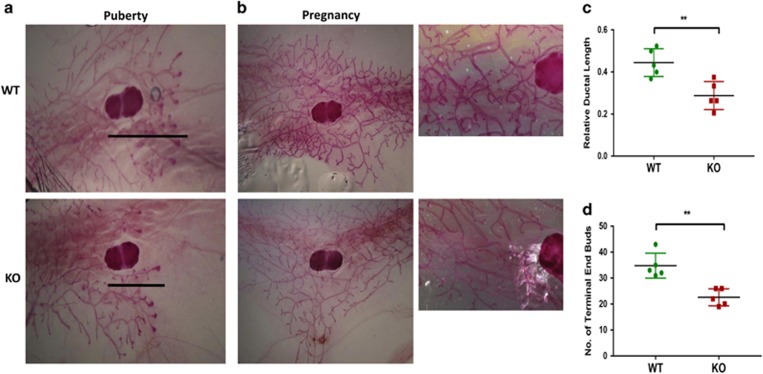
a2V deficiency impairs ductal elongation and bifurcation. Carmine alum staining and quantification of mammary gland whole mounts from puberty and pregnancy. (**a**) In pubertal mice, a2V-KO glands show delayed ductal outgrowth compared with WT. Lines represent ductal migration from the beginning of lymph node to the tip of furthest migrating duct. (**b**) Whole mounts from early pregnant (pregnant day 4.5) mammary gland demonstrate reduced tertiary branching and elongation in a2V-KO mice. Images were taken with x2.5 (left panels) and x20 (right panels) objective. (**c**) Quantification of relative ductal length was measured as a difference between whole length of the gland (from nipple end to tip of ductal tree) and ductal migration (from beginning of lymph node to the tip of ductal tree). (**d**) Quantification of number of TEBs (end nodes of ducts) in WT and KO mice. Data represent mean±S.E., *n*=6. **P≤0.01

To understand the reason behind lactation defects, we investigated the impact of a2V deficiency on mammary gland architecture through whole-mount analysis. During normal pubertal mammary gland development, numerous ducts with terminal end buds (TEBs) elongate within the stromal fat pad at 5 weeks (puberty), developing side branches and filling the mammary fat pads by 10 weeks (virgin).^31^ At puberty, a2V-KO mice displayed a significant decrease in relative ductal length when compared with WT mice as visualized by the extent of ductal growth beyond the lymph node (Figures 4a and c). Next, we modified a previously described method to quantify efficiently mammary gland morphogenesis (Supplementary Figure S2a). Quantification of the ductal tree revealed a decrease in the number of both TEBs and ductal branches in a2V-KO mice (Figure 4d and Supplementary Figure S2b). Whole mounts from early pregnant (pregnant day 4.5) mammary glands of a2V-KO mice showed decreased ductal elongation, bifurcation and tertiary branching (Figure 4b). Taken together, these results suggest that a2V is a key regulator of mammary gland morphogenesis and loss of a2V results in delayed ductal development.

### a2V controls luminal integrity of mammary epithelium

**Figure 5:**
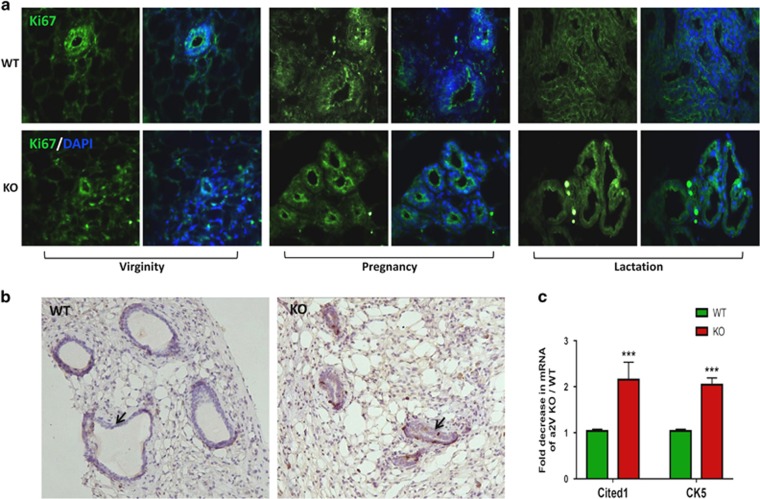
a2V ablation leads to disintegrated mammary epithelium. Inguinal mammary glands from WT and KO mice were evaluated for epithelial lumen integrity and proliferation. (**a**) Immunofluorescence staining of proliferation marker Ki67 (green) in mammary epithelia of WT and KO mice from virginity, pregnancy and lactation stages. Nucleus is stained by DAPI (diamidino-2-phenylindole; blue). (**b**) Representative images from immunohistochemistry highlighting constricted lumen (black arrows) in virgin a2V-KO mice processed for incorporation of 5-bromo-2′-deoxyuridine (BrdU). Brown staining – 3,3′ diaminobenzidine (DAB). Original magnification: × 200. (**c**) Quantitative real-time-PCR (qRT-PCR) shows fold decrease in mRNA expression levels of mammary epithelial cell markers Cited 1 and Keratin 5. Before fold-change calculation, the values were normalized to endogenous control glyceraldehyde 3-phosphate dehydrogenase (GAPDH). Data represent mean±S.E., *n*=6. ***P≤0.001

Multicellular lumen formation involves invagination of apical cells and rearrangement of underlying cells. During mammary ductal formation, the apically oriented luminal epithelial cells undergo extensive cell proliferation and rearrangement.^32^ Considering the role of V-ATPase in luminal acidification and cell proliferation,^29, 33^ we examined epithelial cell proliferation by immunofluorescence staining with proliferation marker Ki67. We found no difference in the cellular expression of Ki67 in WT and a2V-KO mice (Figure 5a). Similarly, WT and KO mice displayed comparable proliferation rate as seen by BrdU incorporation. However, both Ki67 and BrdU staining revealed a constricted lumen and disintegrated mammary epithelia in KO mice. (Figure 5b, black arrows). Further, we assessed the gene expression of Cited 1 and Keratin 5, which are key markers of mammary epithelial cell population.^34^ Expression of both Cited 1 and Keratin 5 was significantly downregulated in a2V-KO mice (Figure 5c) owing to the fact that KO mice have less epithelial content in general and decreased myoepithelial cells in particular as Keratin 5 marks myoepithelial cells in the mammary gland. Taken together, these results suggest that a2V controls mammary epithelial content and architecture.

### Loss of a2V leads to aberrant activation of Notch signaling

**Figure 6:**
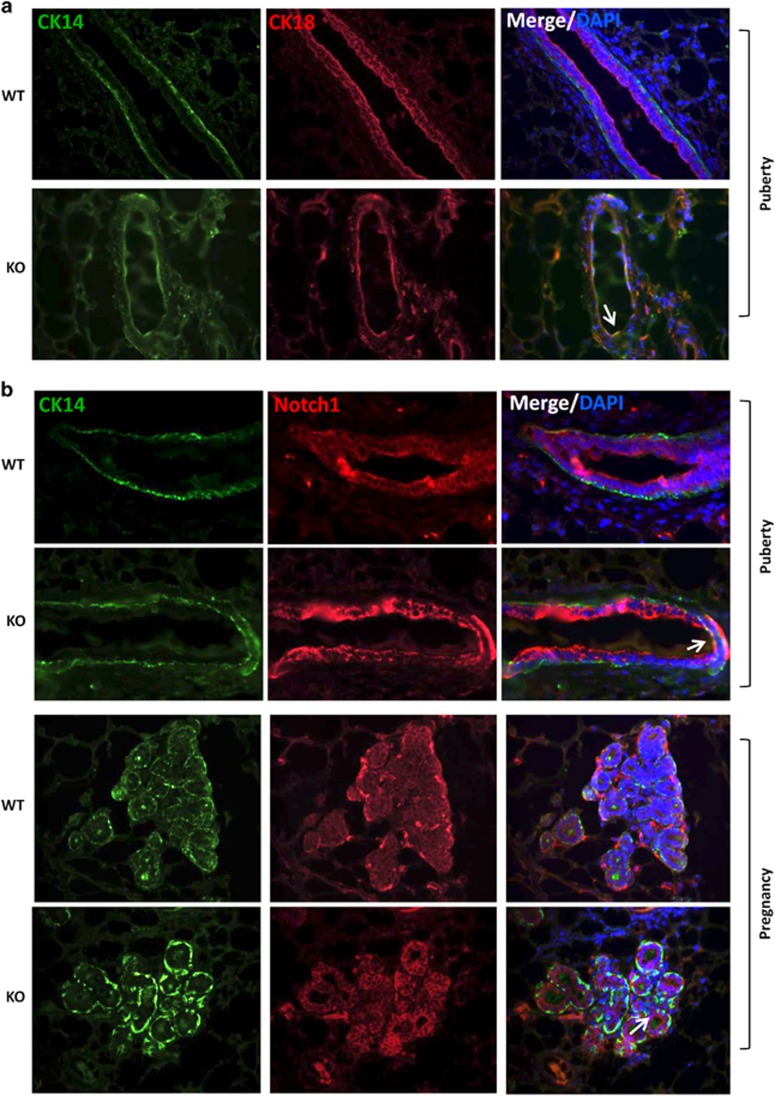
Loss of a2V leads to aberrant activation of Notch in luminal epithelial cells. Mammary glands of WT and KO mice were evaluated for distinct epithelial cell populations. (**a**) Immunofluorescent double staining for basal epithelial cells (CK14 – green) and luminal epithelial cells (CK18 – red) in pubertal mice. (**b**) Expression of NICD (red) costained basal epithelial marker (CK14 – green) in pubertal and pregnant mice by immunofluorescence reveals aberrant activation of Notch 1 in luminal epithelial cells (white arrows). Nucleus was stained with DAPI (diamidino-2-phenylindole; blue)

Notch pathway has been implicated in stem cell maintenance and lineage commitment during mammary gland development. Specifically, Notch signaling drives luminal cell differentiation.^7, 9^ We previously reported that a2V inhibition in breast cancer cell lines affects notch receptor processing and results in aberrant activation of the Notch pathway.^26^ Our finding that a2V is expressed on the plasma membrane of luminal cells led us to investigate the expression of Notch 1 in a2V-KO mice. First, we performed double immunofluorescence for luminal epithelial marker CK18 and basal epithelial marker CK14. The luminal and basal epithelia in WT glands were clearly demarcated, whereas in the KO mice, we observed disintegrated glands with cells expressing both CK18 and CK14, suggesting defective lineage commitment. As expected, WT mice showed distinct exclusive expression pattern of luminal and basal cells. However, we found the presence of CK14 CK18 double-positive cells suggesting lineage commitment defect (Figure 6a, white arrow). Similarly, costaining with Notch 1 and basal epithelial marker CK14 revealed an increase in Notch and also an associated increase in Notch^+^ luminal cells expressing both Notch 1 and CK14 (Figure 6b, white arrows). Confocal microscopy confirmed an increase in Notch signaling as seen by the expression of NICD, the main downstream mediator of Notch pathway (Supplementary Figure S3). A recent report suggested that increase in Notch luminal cells lead to a blockage in lineage commitment of into myoepithelial cells,^9^ connecting our findings of Notch^+^ luminal cell increase to decreased Keratin 5 and lactation defects. Taken together, these results suggest that a2V in addition to directly impacting mammary epithelium also modulates Notch signaling, thereby indirectly controlling epithelial cell fate decisions.

### a2V deficiency results in aberrant activation of TGF*β* pathways

**Figure 7:**
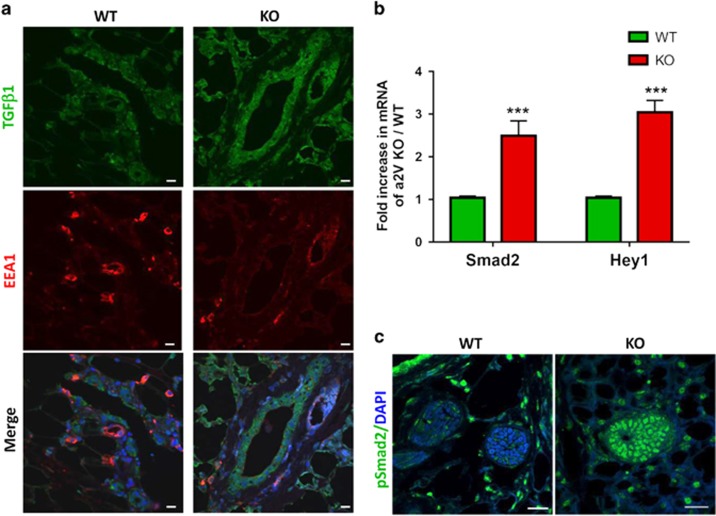
a2V ablation impairs early endosomal trafficking resulting in an increase of TGF-*β* signaling. Inguinal mammary glands from virgin WT and KO mice were evaluated for the status of TGF-*β* and its downstream effectors. (**a**) Localization of TGF-*β* (green) and early endosome marker EEA1 (red) in WT and KO mice by immunofluorescence. Nucleus was stained with DAPI (diamidino-2-phenylindole; blue). Scale bars: 10 *μ*m. (**b**) Fold increase in mRNA expression levels of Notch target gene *Hey1*, and TGF mediator Smad2 were assessed by quantitative real-time-PCR (qRT-PCR). Prior to fold-change calculation, the values were normalized to endogenous control glyceraldehyde 3-phosphate dehydrogenase (GAPDH). Data represent mean±standard error, *n*=6. ***P≤0.001. (**c**) Confocal microscopy for nuclear localization of pSmad2 (green) in pubertal mice. Nucleus was stained with DAPI (blue). Scale bars: 20 *μ*m

Notch and TGF-*β* signaling are important during mammary morphogenesis and aberrant activation of these pathways retards ductal growth.^11, 35, 36^ Furthermore, both Notch and TGF-*β* exhibit similar patterns of expression, spatiotemporal regulation and have functional synergism in the mammary gland.^15^ To investigate the status of TGF-*β* signaling in a2V-KO mice, we costained mammary gland tissue sections for TGF-*β* and early endosomal marker EEA1. Similar to Notch, the expression of TGF-*β* was higher in a2V-KO mice. EEA1 showed a decrease in KO mice, suggesting that the loss of V-ATPase results in impaired acidification and thereby affects endosomal pathway (Figure 7a). We next evaluated the expression of Smad2, the key effector of TGF-*β* and Hey1, a common mediator of both Notch and TGF-*β* pathways, as well as a regulator of the mammary epithelial marker Cited 1.^37^ Expression of both Hey1 and Smad2 was increased in a2V-KO mice compared with WT mice (Figure 7b). Confocal microscopy confirmed the translocation of phosphorylated Smad2 in the nucleus of mammary epithelial cells in a2V-KO mice (Figure 7c). Taken together, these results demonstrate the involvement of Notch and TGF-*β* pathways in a2V-mediated pubertal mammary gland development.

### a2V KD in HMEpCs activates Notch and TGF-*β* signaling

**Figure 8:**
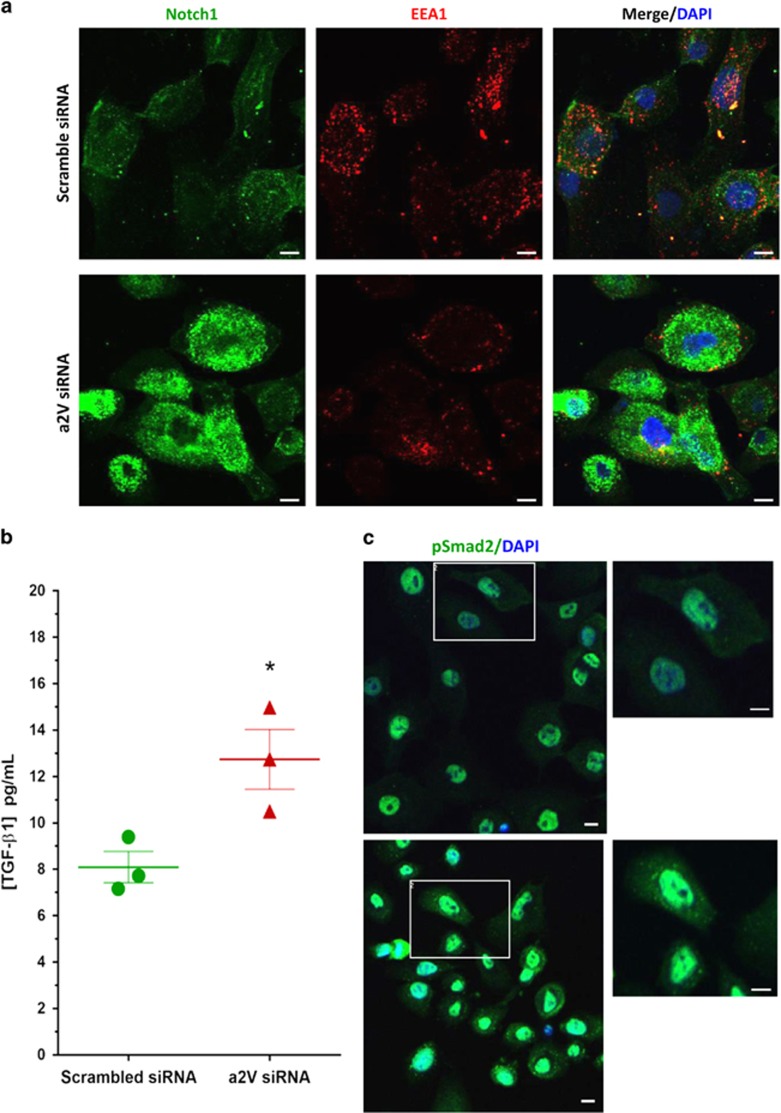
Small interfering RNA (siRNa)-mediated knockdown of a2V in HMEpCs leads to the accumulation of cleaved Notch and secreted TGF-*β*1. HMEpCs were grown in chamber slides and transfected with scrambled control or a2V siRNA and harvested after 48 h of transfection. Cells were fixed, permeabilized and processed for confocal immunofluorescence microscopy. (**a**) Double immunofluorescence staining of Notch 1 intracellular domain (green) with early endosome marker EEA1 (red). Nucleus was stained with DAPI (diamidino-2-phenylindole; blue). Scale bars: 10 *μ*m. (**b**) HMEpCs were seeded in 96-well plates and treated with scramble control or a2V siRNA. Cell supernatant was collected and secreted TGF-*β* was measured by enzyme-linked immunosorbent assay (ELISA) and expressed as pg/ml. Data represent mean±S.E., *n*=3. *P≤0.05. (**c**) Following siRNA-mediated knockdown of a2V, HMEpCs were processed for confocal microscopy for nuclear localization of pSmad2 (green). Nucleus was stained with DAPI (blue). Scale bars: 10 *μ*m

To further investigate whether loss of a2V in normal HMEpCs would have an effect on Notch and TGF-*β* signaling, we used the non-transformed HMEpCs. We performed siRNA-mediated a2V KD in HMEpCs. The efficiency of a2V KD following treatment with gene-specific siRNA was confirmed by RT-PCR (Supplementary Figure S4a). Consistent with our results from the *in vivo* mouse model, we found that a2V KD disrupted endosomal distribution and resulted in increased NICD expression in HMEpCs (Figure 8a). ELISA for TGF-*β* confirmed its increased secretion following a2V KD (Figure 8b). We obtained similar results with TGF-*β* ELISA upon treatment of HMEpCs with bafilomycin, a well-characterized inhibitor of V-ATPase (Supplementary Figure S4b). In line with our results from the mouse mammary gland model, confocal microscopy of a2V KD in HMEpCs revealed an increase in nuclear translocation of pSMAD2 associated with the activation of TGF-*β* pathway (Figure 8c). These findings show that a2V-ATPase modulates Notch and TGF-*β* pathways during normal human mammary epithelial development.

## Discussion

**Figure 9:**
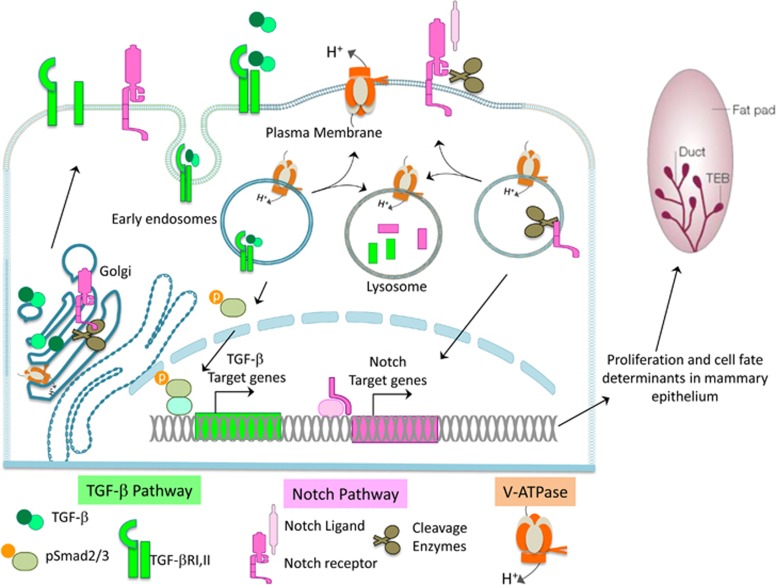
a2V regulates Notch and TGF-*β* signaling in mammary gland through its role in endolysosomal acidification. The V-ATPase (orange) is responsible for acidic pH maintenance in intracellular vesicles like Golgi, endosomes and lysosomes and on the plasma membrane. During canonical Notch Signaling (pink), Notch receptor is cleaved in Golgi and translocated to the plasma membrane where further cleavage of the receptor occurs in response to ligand binding. Finally, NICD is cleaved in the endosomes translocating it to the nucleus to activate Notch target genes. Similarly, TGF-*β* (green) protein is glycosylated in the Golgi and complexed to latent membrane proteins to form mature TGF-*β*, which is secreted into the extracellular space. The binding of TGF-*β* to its receptor (TGF-*β*R) results in endocytosis and phosphorylation of Smad2, which in turn activates TGF-*β* target genes. Both Notch and TGF-*β* pathway mediators depend on V-ATPase-mediated acidification for cleavage and activation of pathway mediators by acid-dependent enzymes, sustenance of basal signaling by recycling endosomes and degradation of signaling molecules in lysosomes. Therefore, manipulation of V-ATPase activity results in dysregulated signaling, which in turn effects mammary epithelial cell fate and proliferation

In this study, we identified a novel role of a2V in mammary gland development. a2V promotes ductal morphogenesis and lactation during mammary gland development. The functional effects of a2V in mammary glands are mediated, in part, by Notch and TGF-*β* pathways. Our results indicate that a2V-dependent acidification of the Golgi and endolysosomal pathways is critical for activation, maturation and degradation of Notch and TGF-*β* pathway mediators. In summary, the V-ATPase regulates developmental signaling pathways and thereby influences mammary epithelial cell fate (Figure 9).

Ductal bilayer of the mammary gland in virgin adult mice is composed of inner luminal epithelial cells and outer layer of myoepithelial cells surrounded by the basement membrane. Towards the tip of invading ducts are structures called TEB that consist of highly proliferative epithelial cap cells followed by epithelial body cells on the interior.^38^ During the formation of ductal lumen, apoptosis of the central body cells occurs to form the lumen, whereas the rest of the body cells differentiate into luminal epithelial cells, giving rise to the ductal tree. During pregnancy, luminal epithelial cells rapidly proliferate, forming lobular alveoli that are involved in milk secretion at parturition.^1^

V-ATPase is a multisubunit proton pump responsible for controlling the intracellular and extracellular pH of cells. We found abundant expression of a2V in both luminal and myoepithelial cells of mammary ducts. Our current results point to the previously elucidated role of a2V in regulating matrix metalloproteinase-9 and extracellular acidification in ovarian cancer.^39^ In the mammary gland too, ductal elongation requires invasion into surrounding stroma.^32^ This invasion is brought about, in part, by the extracellular matrix-degrading enzymes such as matrix metalloproteinase-9,^40^ demonstrating a direct involvement of a2V in lumen formation and ductal invasion.

In the developing mammary gland, a2V expression gradually increased from puberty to lactation and then decreased during involution. The loss of a2V results in glands with reduced ductal length and diameter with branching defects. a2V is therefore an important molecule at the leading edge of epithelial cells during ductal elongation and branching. We observed robust expression of a2V in the lactation stage and a2V deficiency resulted in lactation defects. However, the exact role of a2V in milk secretion by glandular epithelial cells and ejection by myoepithelial cell contractions remains to be investigated.

The role of acidification during mammary gland involution is well characterized. Following lactation, the mammary gland undergoes a weaning phase or involution, which involves massive and carefully orchestrated apoptosis of the epithelial cells. At this time, the gland remodels to its prepregnancy state. Reports suggest that lysosome-mediated cell death has a major role in macrophage recruitment and clearance of debris during involution.^41^ Further, key molecules such as cathepsins are released by lysosomes to sense the accumulated milk and trigger apoptosis.^42, 43^ V-ATPase could be crucial during this phase, considering its role in the activation and release of cathepsins in response to cell death stimuli.^44^

Notch signaling have an important role during development and tumorigenesis. Accumulating evidence supports the association of Notch signaling in stem cell maintenance and luminal cell decision during mammary gland development.^5^ Further, the aberrant upregulation of Notch was shown to impair gland morphogenesis through dysregulated signaling.^9, 36^ In this context, observations of enhanced Notch signaling and Notch^+^ luminal cells in response to a2V inhibition suggest that in addition to its direct involvement in mammary morphogenesis, a2V has an indirect role in mammary development through the regulation of signaling pathways.

The Notch pathway exerts its effects in development and cancer through interaction with other signaling pathways such as Wnt and TGF-*β*.^45^ Notably, Notch and TGF-*β* have similar patterns of expression and spatiotemporal regulation in several organ systems.^15^ The TGF-*β* pathway is a crucial regulator of epithelial-to-mesenchymal transition during cellular differentiation of mammary epithelial cells. Reports show that similar to Notch, aberrant TGF activation in the mammary gland impairs morphogenesis.^13, 46^ Importantly, human mutations in a2V gene cause autosomal-recessive Cutis Laxa syndrome where patients present with a decreased amount of extracellular matrix proteins such as collagen, resulting in wrinkly skin phenotype. Recent reports point to a glycosylation defect in Cutis Laxa, resulting in elevated TGF-*β* signaling in these individuals with a2V mutations. Notably, it has been shown that in Cutis Laxa patients, mutations in both a2V and latent membrane protein 4 stabilizes TGF-*β* receptors by preventing their endocytosis and lysosomal degradation resulting in enhanced signaling.^47, 48^ In line with these reports, we find that a2V-KO mice show activation of TGF-*β* pathway. In our recent experiments, we found that tumor xenografts from a2V-KO mice have an increased growth rate of injected mammary tumors. These mice also displayed a reduction of total collagen because of impaired glycosylation (Gajendra Katara *et al.*, unpublished data).

Mechanistically, the retention of Notch in endosomal vesicles accelerates the protein's cleavage and intensifies Notch signaling.^49^ Furthermore, the maturation of both Notch and TGF-*β* by glycosylation in Golgi vesicles is a requirement for activation of signaling.^50^ We previously reported that in breast cancer, a2V inhibition enhances Notch signaling by blocking lysosomal and autophagic degradation of Notch receptor.^26^ Similarly, lack of autophagy was shown to accumulate NICD and Wnt pathway mediator *β*-catenin in *Drosophila* and human cancer cells, thereby enhancing Notch and Wnt Signaling.^51, 52^ Recent reports also highlight the collective roles of autophagy and TGF-*β* in bovine mammary epithelial cells.^53^ V-ATPase is crucial for the integrity of both endolysosomal and autophagic vacuoles and disruption of V-ATPase activity has distinct and context-dependent effects on signaling by Wnt, Notch and TGF-*β* pathways. Indeed, both positive and negative outcomes of endolysosomal pathway on major developmental signals such as Notch, TGF-*β* and Wnt have been highlighted before.^16, 54, 55^ We note that while a2V in particular emerges as a key regulator of Notch and TGF pathways in mammary gland development, the vacuolar-ATPase, in general, regulates signaling pathways in development and disease and therefore will have a tremendous impact in physiology and pathophysiology. Finally, as Notch, Wnt and TGF-*β* pathways share similar expression patterns and cellular functions during both mammary gland development and breast cancer,^56^ further studies are warranted to elucidate their regulation by the endolysosomal vesicles.

## Materials and Methods

### Generation of a2V^−/−^ mice

All animal procedures were conducted in compliance with the guidelines of IACUC at Rosalind Franklin University. a2V conditional-targeting vector was generated by inGenious Targeting Laboratories (Ronkonkoma, NY, USA). Briefly, a 1.38 kb region spanning exons 12–14 of the mouse a2V (gene name ATP6V0a2) was retrieved into a pLenti6 vector. A LoxP-FRP neo cassette was then introduced upstream of exon 12 along with restriction enzymes (*Spe*I and *Nsi*I). Targeted embryonic stem cell clones with LoxP a2V (a2Vf/+) allele were screened and injected into albino C57BL/6 blastocysts at Charles River Laboratories (Wilmington, MA, USA). The resulting chimeras were bred with C57BL/6 to produce heterozygous a2Vf/+ mice, and intercrossed to obtain homozygous a2Vf/f. MMTV-Cre transgenic line D mice were purchased from the Jackson Laboratories (Bar Harbor, ME, USA; stock no. 003553) and have a mixed B6129F1, FVB background. MMTV-Cre mice were backcrossed with a2Vf/f mice to generate MMTV-Cre/a2Vf/f mice (referred to as a2V^−/−^ or KO) with a2V-specific KO in mammary epithelial cells. 'WT' littermates with the genotype a2Vf/f or MMTV-Cre/a2V^+/+^ were used as controls (referred to as a2V^+/+^ or WT). For routine genotyping, mouse-tail DNA tissue was prepared using Extract-N-Amp Tissue PCR Kit (No. XNAT2-1KT, Sigma-Aldrich, St.Louis, MO, USA). To detect MMTV-Cre transgenic insert, generic Cre PCR was performed following the Jackson Laboratory protocol. For a2V-loxP genotyping: forward primer, 5′-AGGGTGGTGTCCTTTCACTCT-3′ and reverse primer, 5′-ATCCCCAGGATCCACGCAT-3′ were used. Agarose gel electrophoresis produced 184 and 245 bp bands for a2V+ and a2Vf, respectively.

### Cell culture and reagents

HMEpCs were obtained and grown in mammary epithelial cell growth medium (Cell Applications, San Diego, CA, USA). Cells were maintained in a humidified incubator at 37 °C and 5% CO_2_ atmosphere. For V-ATPase inhibition, bafilomycin A1 (Millipore, Darmstadt, Germany) was dissolved in DMSO and used at the indicated concentrations.

### Whole-mount morphological analysis

To study gland development, inguinal mammary glands were collected from females in the following stages: puberty (5-week-old), virginity (10-week-old), pregnancy (embryonic day E14.5), lactation (nursing day 14) and involution (postweaning day 3). Mammary whole-mount analysis was performed as described previously.^57^ Glands were dissected and spread out on a glass slide, soaked overnight in Carnoy's fixative, washed with 70% ethanol and gradually rehydrated with water and stained with Carmine Alum (both from VitroView Mammary Gland Whole-mount Stain Kit; Genecopoeia, Rockville, MD, USA). Stained glands were cleared in ethanol and xylene and mounted with Permount (Thermo Fisher Scientific, Waltham, MA, USA). Pictures were taken with a MZ75 light microscope (Leica, Wetzlar, Germany). Ductal elongation and bifurcation was quantified as reported previously.^58^

### Growth-curve analysis

For growth-curve analysis, female MMTV-Cre/a2V^f/f^ mice aged 6–8 weeks were mated with male a2V^f/f^ mice. Pups from litter sizes 5–8 were weighed on day 5 and at two-day interval from day 6 onwards. Further, body weights and mammary glands weights were measured and compared between pregnant (E14.5) a2V WT and KO mice.

### Immunohistochemistry

Inguinal mammary glands were dissected and fixed in 4% paraformaldehyde overnight followed by 30% sucrose for 24 h. Fixed tissues were embedded in OCT (Tissue-Tek, Sakura Finetek, Torrence CA, USA) and frozen. Sections of 5 *μ*m thick were cut with a cryostat and transferred to charged slides. For antigen retrieval, sections were boiled in sodium citrate buffer (pH=6). Immunohistochemical staining of Notch 1 (Antibody clone C-20, Santa Cruz Biotechnology, Dallas, Texas, USA), and anti-a2V (clone 2c1 described previously in ^39^ was carried out using a method based on horseradish peroxidase-labeled polymer (EnVision+Dual Link System-HRP; Dako, Carpinteria, CA, USA) according to the manufacturer's protocol. The sections were counterstained with Mayer's hematoxylin and mounted in Faramount aqueous mounting medium (Dako) and imaged by light photomicroscopy (Carl Zeiss, Weesp, The Netherlands). For BrdU incorporation assay, adult virgin mice (10 weeks old) were injected intraperitoneally with BrDu labeling reagent (Thermo Fisher Scientific) at 70 mg/kg for 2 h. Mammary glands were then collected and processed for immunohistochemistry with VitroView *In Situ* BrdU Detection Kit (Genecopoeia, Rockville, MD, USA).

### Immunofluorescence microscopy

Mammary gland cryosections were processed as described above for immunohistochemistry and evaluated by confocal microscopy. HMEpCs were plated in 8-well chamber slides (Nunc, Thermo Fisher Scientific) at 1 × 10^4^ cells per well and were allowed to adhere overnight. Cells were washed with PBS, fixed for 15 min with 4% paraformaldehyde, and permeabilized with 0.1% Triton X-100 for 10 min. For both tissues and cells, nonspecific sites were blocked for 1 h at room with 3% BSA, washed in PBS-T and incubated at 4 °C overnight with primary antibodies: Notch 1 (antibody clone EP1238Y, ab52627), EEA1 (ab70521), TGF-*β*1 (ab66043), pSmad2 (ab188334) and Ki67 (ab15580) were from Abcam, Cambridge, MA, USA; cytokeratin 18 (Fitzgerald, North Acton, MA, USA; 70R-30585) and cytokeratin 14 (Thermo Fisher Scientific; MA5-11599). After primary antibody incubation, slides were washed three times with PBS and incubated with secondary antibodies: Alexa Fluor 488 goat anti-rabbit (A-11008) and Alexa Fluor 594 donkey anti-mouse (A-21203) (Invitrogen, Thermo Fisher Scientific) for 1 h at room temperature. The stained slides were imaged on an Olympus Fluoview Fv10i confocal microscope (Olympus, Waltham, MA, USA). Analysis was performed using Fv10i Flouview Ver.3.0 software (Olympus).

### Real-time PCR

Total RNA from mammary tissues and HMEpCs was extracted by Trizol Reagent (Ambion, Thermo Fisher Scientific) and quantified using NanoDrop ND-2000 (NanoDrop Technologies, Wilmington, DE, USA). RNA was reverse transcribed into cDNA using First-Strand cDNA Synthesis Kit (Roche Diagnostics, Mannheim, Germany). RT-PCR was performed using StepOnePlus Real-time PCR (Applied Biosystems-Life Technologies) with 10 *μ*l reaction volume with mouse *GAPDH* or human*18srRNA* as the internal reference. Prevalidated Taqman Gene expression primers for V-ATPase ‘a' subunit isoforms *ATP6V0a1*, *ATP6V0a2*, *ATP6V0a3* and *ATP6V0a4*; Notch pathway genes *Notch 1*, *Hey1*; *TGF-β1*, *pSmad2*, mammary proliferation markers *Areg*, *Cited 1*, lactation marker *β*-*casein* and internal control *GAPDH* and *18srRNA* were all purchased from Applied Biosystems. Universal Fast PCR Master Mix Reagent (Applied Biosystems, Thermo Fisher Scientific) was used for qPCR amplification of the cDNA. Relative gene expression was calculated using the ΔCt method. Fold change was calculated using the ΔΔCt method.

### RNA interference

siRNA pools specific for a2V and scrambled control were purchased from Origene (Rockville, MD, USA). Cells were plated in 12-well plates or 8-well chamber slides and allowed to adhere overnight. Cells were transfected with siRNA (final concentration, 10 nM) using lipid-mediated transfection with Lipofectamine 3000 (Invitrogen) according to the manufacturer's instructions. For all experiments, data were collected 48 h post-transfection.

### ELISA

The amount of secreted TGF-*β*1 by HMEpC cells was quantified using the Quantikine human TGF-*β*1 ELISA Kit (R&D Systems, Minneapolis, MN, USA) according to the manufacturer's protocol. Briefly, 1 × 10^5^ cells were plated onto 48-well plates, and treated with a2V siRNA for 48 h or 1 *μ*M bafilomycin. Culture media supernatants were analyzed for TGF-*β*1 secretion by relative absorbance reading at 450 nm using a Multi-Detection Micro Plate Reader (Synergy HT; Biotek Instruments, Winooski, VA, USA). Values are expressed as secreted TGF-*β* 1 pg/ml per well.

### Statistical analysis

All experiments were conducted in triplicates with at least six mice per experiment. The results are expressed as mean values of S.E.M. Groups were compared by Student's *t-* test, one-way or two-way ANOVA followed by appropriate post-tests using GraphPad Prism 7.0 software (Graphpad Software, LaJolla, CA, USA). Differences between groups were considered statistically significant at *P*<0.05.

## Abbreviations

a2V: a2V-ATPase, a2 isoform of the vacuolar-ATPase
Hey1: hairy/enhancer-of-split related with YRPW motif
HMEpC: human mammary epithelial cell line
MMP: matrix metalloproteinase
NICD: Notch intracellular domain
RBP-Jk: recombination signal binding protein for immunoglobulin
TGF-*β*: transforming growth factor-*β*
