# Supplementary material for: The V-ATPase a2 isoform controls mammary gland development through Notch and TGF-β signaling
Source: Cell Death Dis. 2016 Nov 3;7(11):e2443–. doi: 10.1038/cddis.2016.347 (PMC5260869; doi:10.1038/cddis.2016.347)
Supplement: Supplementary Figures [file cddis2016347x1.ppt]

## Slide 1
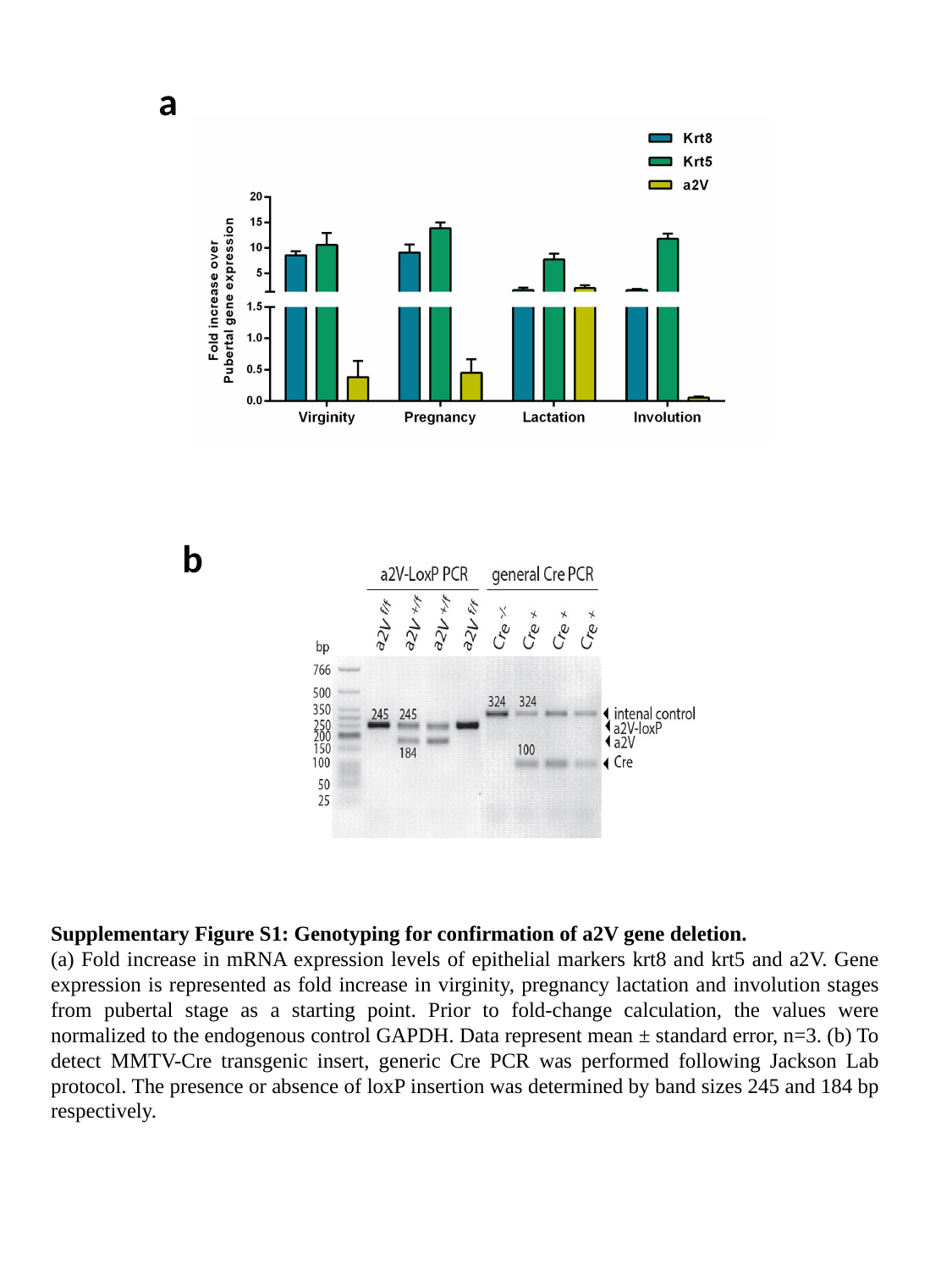

a
b
Supplementary Figure S1: Genotyping for confirmation of a2V gene deletion.
(a) Fold increase in mRNA expression levels of epithelial markers krt8 and krt5 and a2V. Gene expression is represented as fold increase in virginity, pregnancy lactation and involution stages from pubertal stage as a starting point. Prior to fold-change calculation, the values were normalized to the endogenous control GAPDH. Data represent mean ± standard error, n=3. (b) To detect MMTV-Cre transgenic insert, generic Cre PCR was performed following Jackson Lab protocol. The presence or absence of loxP insertion was determined by band sizes 245 and 184 bp respectively.

## Slide 2
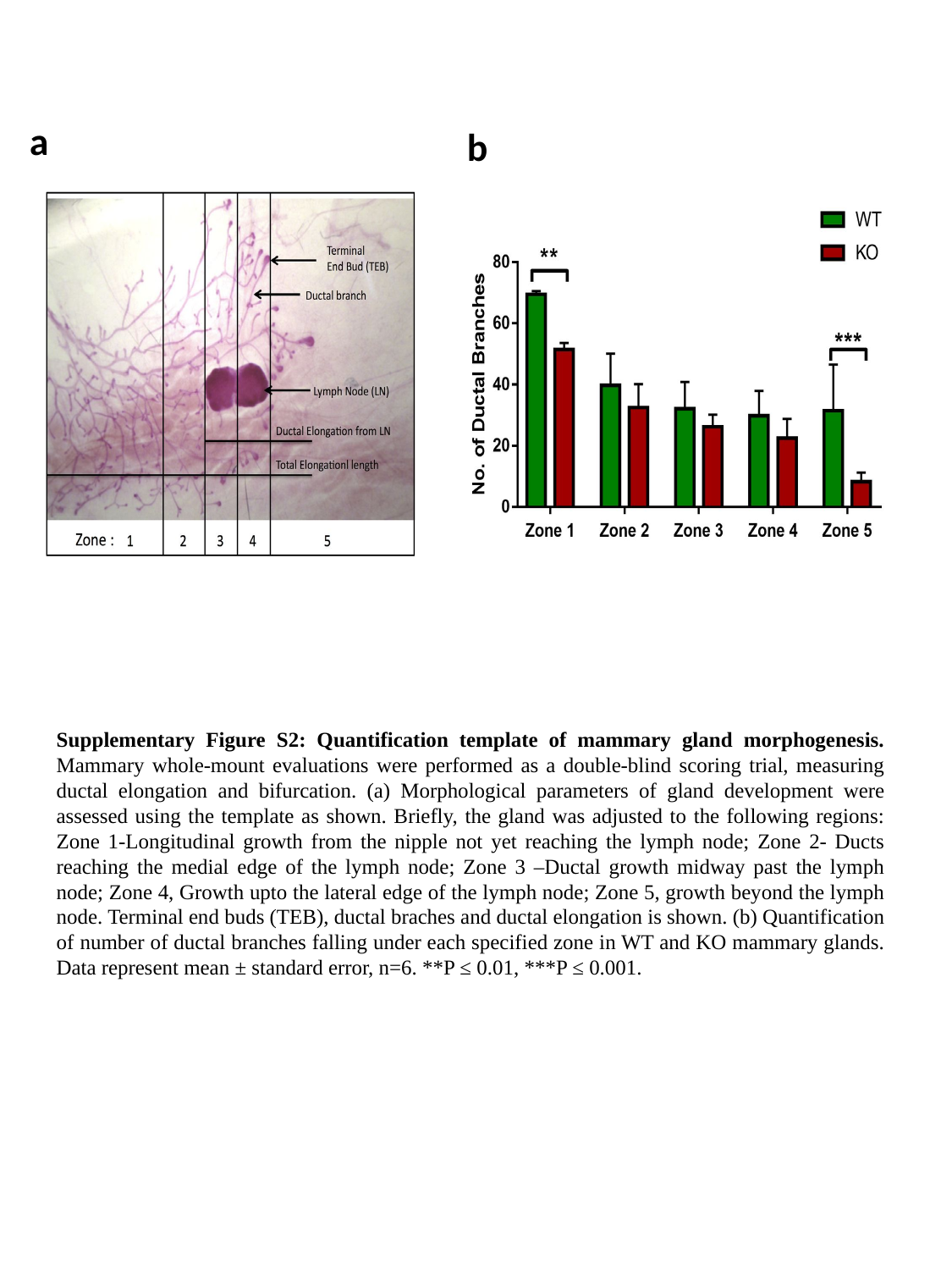

a
b
Supplementary Figure S2: Quantification template of mammary gland morphogenesis. Mammary whole-mount evaluations were performed as a double-blind scoring trial, measuring ductal elongation and bifurcation. (a) Morphological parameters of gland development were assessed using the template as shown. Briefly, the gland was adjusted to the following regions: Zone 1-Longitudinal growth from the nipple not yet reaching the lymph node; Zone 2- Ducts reaching the medial edge of the lymph node; Zone 3 –Ductal growth midway past the lymph node; Zone 4, Growth upto the lateral edge of the lymph node; Zone 5, growth beyond the lymph node. Terminal end buds (TEB), ductal braches and ductal elongation is shown. (b) Quantification of number of ductal branches falling under each specified zone in WT and KO mammary glands. Data represent mean ± standard error, n=6. **P ≤ 0.01, ***P ≤ 0.001.

## Slide 3
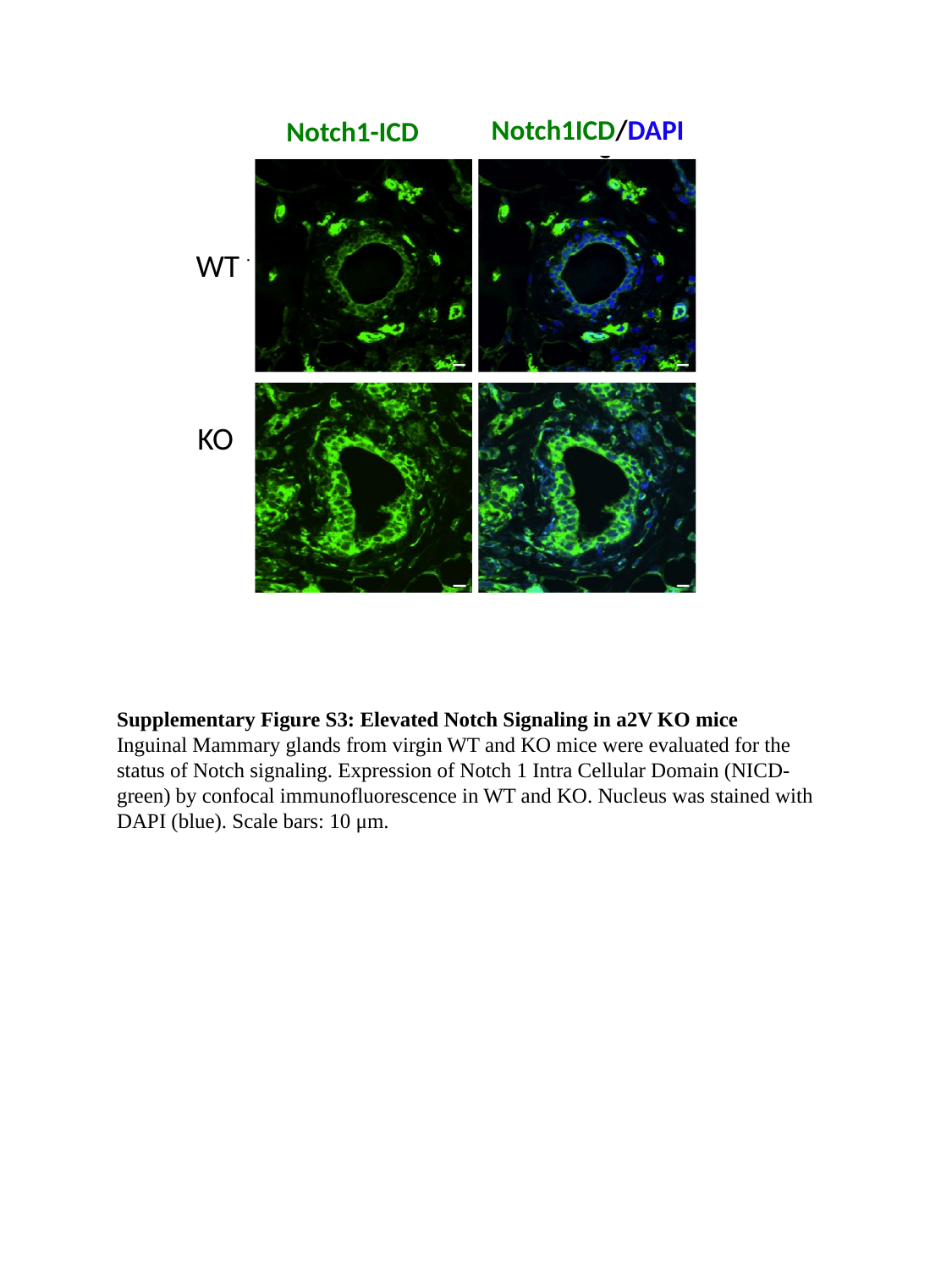

Notch1ICD/DAPI
Notch1-ICD
WT
KO
Supplementary Figure S3: Elevated Notch Signaling in a2V KO mice
Inguinal Mammary glands from virgin WT and KO mice were evaluated for the status of Notch signaling. Expression of Notch 1 Intra Cellular Domain (NICD-green) by confocal immunofluorescence in WT and KO. Nucleus was stained with DAPI (blue). Scale bars: 10 μm.

## Slide 4
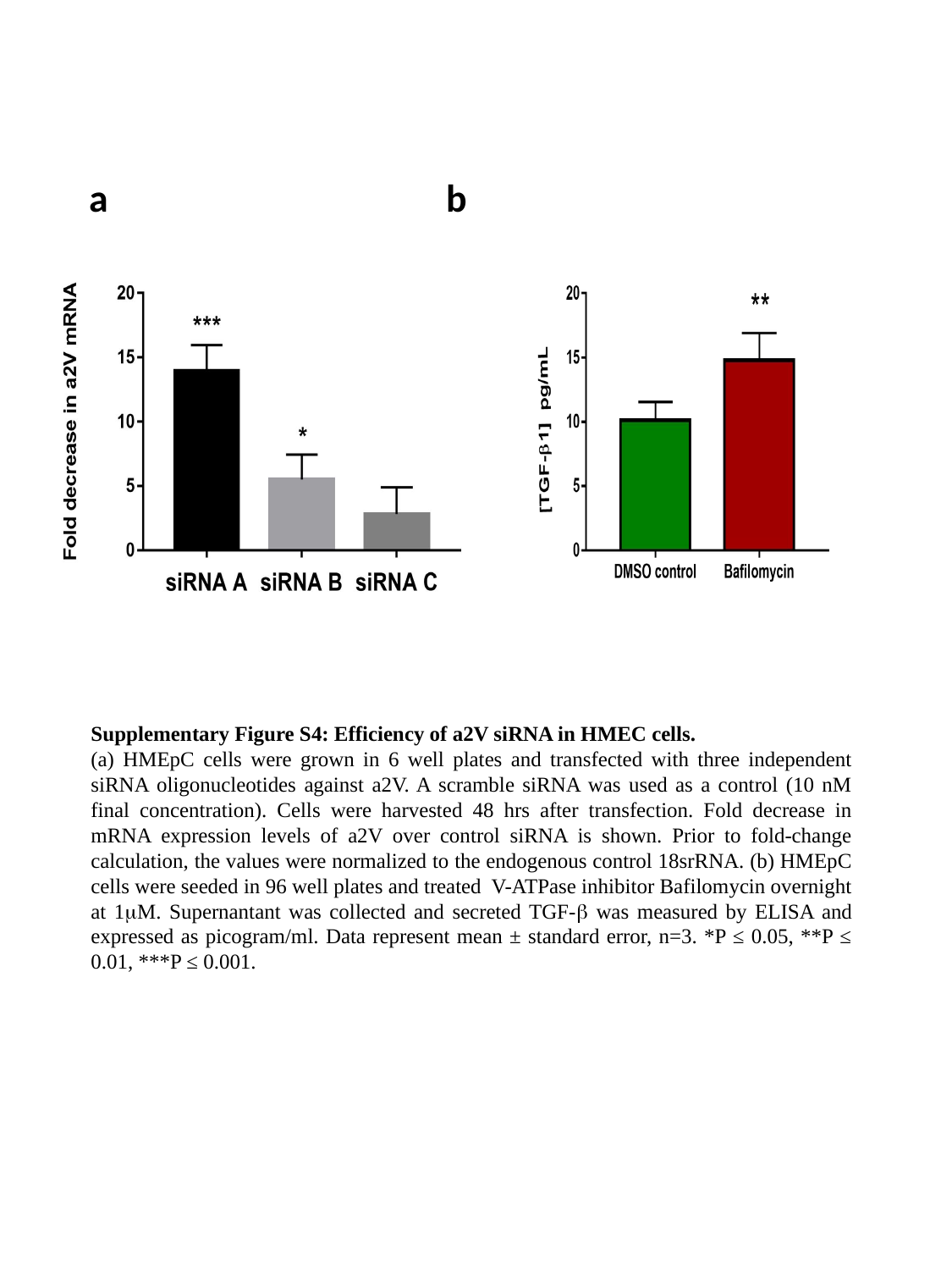

a
b
Supplementary Figure S4: Efficiency of a2V siRNA in HMEC cells.
(a) HMEpC cells were grown in 6 well plates and transfected with three independent siRNA oligonucleotides against a2V. A scramble siRNA was used as a control (10 nM final concentration). Cells were harvested 48 hrs after transfection. Fold decrease in mRNA expression levels of a2V over control siRNA is shown. Prior to fold-change calculation, the values were normalized to the endogenous control 18srRNA. (b) HMEpC cells were seeded in 96 well plates and treated V-ATPase inhibitor Bafilomycin overnight at 1μM. Supernantant was collected and secreted TGF-β was measured by ELISA and expressed as picogram/ml. Data represent mean ± standard error, n=3. *P ≤ 0.05, **P ≤ 0.01, ***P ≤ 0.001.
